# Supplementary material for: Direct Photochemical Synthesis of Substituted Benzo[b]fluorenes
Source: Org Lett. 2024 Nov 25;26(48):10364–8. doi: 10.1021/acs.orglett.4c03978 (PMC11629382; doi:10.1021/acs.orglett.4c03978)
Supplement: Supplementary file 2 — ol4c03978_si_002.zip [file ol4c03978_si_002.zip › FID for Publication/5d/20240424_RC-2-C-5-69_01/plots/RC-2-C-5-69_gCOSY_20240424_01_plot01.pdf]

**RC-2-C-5-69**

| | | | | | | | |
|---|---|---|---|---|---|---|---|
| Location | **3** | Pulse sequence | **gCOSY** | Temperature | **25** | Suboperator | **RuairiCrawford** |
| Date collected | **2024-04-24** | Solvent | **dmso** | Spectrometer | **Agilent-NMR-vnmrs500** | Operator | **MBaumann** |

**SAMPLE**

| | |
|---|---|
| date | **Apr 24 2024** |
| solvent | **dmso** |
| sample | **20240424_RC-2-C-5-69_01** |

**ACQUISITION**

| | |
|---|---|
| sw | **5411.3** |
| at | **0.150** |
| np | **1624** |
| fb | **4000** |
| ss | **32** |
| d1 | **1.000** |
| nt | **4** |

**2D ACQUISITION**

| | |
|---|---|
| sw1 | **5411.3** |
| ni | **400** |
| d2 | **0** |

**PRESATURATION**

| | |
|---|---|
| satmode | **n** |
| wet | **n** |

**TRANSMITTER**

| | |
|---|---|
| tn | **H1** |
| sfrq | **500.035** |
| tof | **271.0** |
| tpwr | **62** |
| pw | **7.200** |

**GRADIENTS**

| | |
|---|---|
| gzlvlE | **5291** |
| gtE | **0.001000** |
| EDratio | **1.000** |
| gstab | **0.000500** |

**DECOUPLER**

| | |
|---|---|
| dn | **C13** |
| dm | **nnn** |

**FLAGS**

| | |
|---|---|
| hs | **nn** |
| sspul | **y** |
| hsglvl | **6348** |

**SPECIAL**

| | |
|---|---|
| temp | **25.0** |
| gain | **40** |
| spin | **0** |

**F2 PROCESSING**

| | |
|---|---|
| sb | **-0.075** |
| sbs | **not used** |
| fn | **2048** |

**F1 PROCESSING**

| | |
|---|---|
| sb1 | **-0.050** |
| sbs1 | **not used** |
| proc1 | **lp** |
| fn1 | **2048** |

**DISPLAY**

| | |
|---|---|
| sp | **65.5** |
| wp | **5411.3** |
| sp1 | **65.5** |
| wp1 | **5411.3** |
| rfl | **-65.5** |
| rfp | **0** |
| rfl1 | **-65.5** |
| rfp1 | **0** |

**PLOT**

| | |
|---|---|
| wc | **216.8** |
| sc | **0** |
| wc2 | **124.5** |
| sc2 | **[illegible]** |
| vs | **120** |
| th | **9** |
| ai cdc av | |

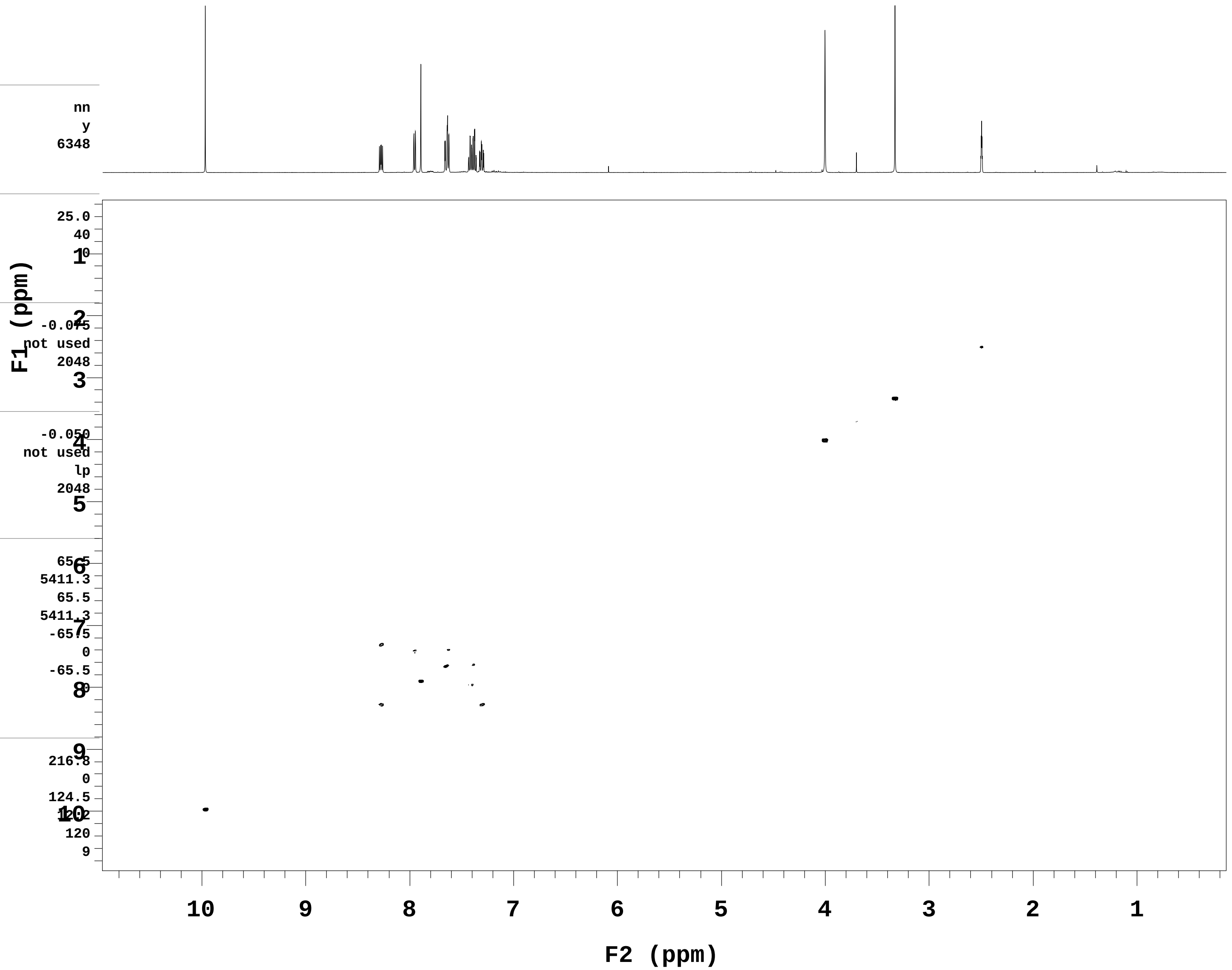


**Plotname:** **RC-2-C-5-69_gCOSY_20240424_01_plot01**

**Data file** /home/data/MBaumann/newucd500/RuairiCrawford/2024/20240424_RC-2-C-5-69_01/RC-2-C-5-69_gCOSY_20240424_01.fid

Plot date 2024-04-24
